# Acceptability of a Peer‐Led Faith‐Based Intervention to Improve Breast, Bowel and Cervical Cancer Screening Uptake Among UK Muslim Women: A Qualitative Evaluation

**DOI:** 10.1111/hex.70935

**Published:** 2026-10-16

**Authors:** Rawand Jarrar, Kathryn A. Robb, Marie K. Murphy, Cerysh Sadiq, Anna Matthews, Fozia Haider, Alex McConnachie, Aasim I. Padela, Floor Christie‐de Jong

**Affiliations:** ^1^ School of Medicine, Faculty of Health Sciences and Wellbeing University of Sunderland Sunderland UK; ^2^ School of Health and Wellbeing, University of Glasgow Glasgow UK; ^3^ Medical College of Wisconsin Milwaukee USA

**Keywords:** acceptability, bowel cancer, breast cancer, cancer screening, cervical cancer, faith, health inequities, Muslim women, participatory research, religious tailoring

## Abstract

**Background:**

Early cancer detection through screening can improve treatment outcomes. However, screening uptake remains lower among some communities, including Muslim women, which puts them at risk of late diagnosis and poor outcomes. In 2020, we worked with 10 Scottish Muslim women to co‐design a peer‐led, faith‐based and culturally tailored intervention to encourage uptake of breast, bowel and cervical cancer screening. The intervention was delivered twice online and qualitatively evaluated. The study was subsequently funded as a feasibility trial, and the intervention was delivered a further 16 times, both online and face‐to‐face. This paper reports findings from a qualitative evaluation exploring participants' perspectives on the acceptability and implementation of the intervention.

**Methods:**

The intervention is a 2‐h workshop comprising four components: (1) a peer‐led cancer screening discussion, (2) a doctor‐led health education session, (3) testimonials from Muslim women, and (4) an Islamic perspective delivered by a female religious scholar. In 2024, women participated in online or in‐person workshops in North‐East England and Scotland. After each workshop, women were invited to participate in focus groups or interviews to provide feedback on the workshop. Qualitative data collection and analysis were guided by the Theoretical Framework of Acceptability. Data were analysed using the Framework Analysis method.

**Results:**

A convenience sample of 36 women was recruited. Participants described the workshop's content as comprehensive and providing new information. The workshop's informal atmosphere and use of different delivery methods enhanced information attainment. They appreciated the use of interpreters, who assisted non‐English speakers. Bringing a medical and Islamic perspective together was novel to participants and made the workshop more impactful. Participants expressed more positive attitudes towards screening after the intervention.

**Conclusion:**

A peer‐led, culturally‐tailored and faith‐based intervention for improving cancer screening uptake for Muslim women was deemed acceptable. However, more work is needed to assess the intervention's effectiveness and impact.

**Patient or Public Contribution:**

The intervention was co‐designed with 10 Muslim women. A public involvement group worked with the research team throughout the study; they provided their views on the intervention, recruitment, data collection and interpretation of findings. Some were trained as peer educators to help deliver the intervention. Two members of the community (FH and CS, who are also listed as authors) were also employed on the project as Community Recruitment Leads, facilitating participant recruitment and contributing to all stages of the research as valued members of the research team, ensuring the community's voice was embedded throughout.

**Abbreviations**
FIT: faecal immunochemical test
GP: general practitioner
NHS: National Health Service
PICE: public involvement and community engagement
TFA: Theoretical Framework of Acceptability
UK: United Kingdom

## 1 Introduction

Although survival following cancer diagnosis has improved in recent years, efforts are needed to minimise the burden of the disease through early detection [1]. In the United Kingdom, the National Health Service (NHS) has breast, bowel, and cervical cancer screening programmes that aim to detect cancer before the appearance of symptoms [2]. Each screening is offered to all women registered with a general practitioner (GP) after they reach the eligible age; breast screening is offered to women aged 50–71 every 3 years [3], and women aged 25–64 years are offered cervical screening [2]. Bowel screening is done by sending a faecal immunochemical test (FIT) kit. In Scotland, women aged 50 to 74 are sent a FIT kit. Until recently, bowel screening was offered to women aged 60–74 in England [4]; the starting age was recently lowered to 50 years [5].

Screening uptake has been below the targets set for the three types of screening [6], with uptake significantly lower among ethnic minorities and people living in deprived areas [7]. Muslims form 6% of the total UK population and predominantly come from minority ethnic backgrounds, with 40% of Muslims living in deprived areas and 2% cannot speak English [8, 9, 10]. London is the region that has the highest religious diversity with Muslims making up 15% of the London population [9]. Previous studies found lower screening attendance among Muslim women compared to the general population [11, 12, 13]. This increases the risk of detecting cancer at later stages, leading to poor treatment outcomes and higher mortality rates. Evidence suggests that each 4‐week delay in cancer treatment can increase the risk of death by 6%–13% [14]. Therefore, it is vital to increase screening uptake among Muslim women.

Barriers to breast, bowel and cervical screening among Muslim women are complex; these include, but are not limited to, emotional barriers, lack of awareness, perceived cancer risk, previous negative experience with screening, language barriers, social stigma and lack of support from the family and community [15, 16, 17]. Interventions that promote Muslim women's screening uptake should be culturally‐tailored and respond to their beliefs, rather than adopting a one‐size‐fits‐all approach [16].

Integrating religious messages in interventions to encourage uptake of cancer screening was found to improve participation among Muslims in the United States, which demonstrates that a faith‐based approach can help overcome barriers to screening [18, 19, 20]. Additionally, participatory approaches, where community members are involved in the design and delivery of interventions, can engage communities in research projects and reduce disparities for ethnic minorities [21, 22]. Participatory approaches consider community members as equal partners in research working alongside academic researchers to integrate scientific knowledge with lived experiences [22, 23]. This results in better health outcomes, especially among minority populations [22, 23].

A few studies implemented faith‐based interventions to improve breast, bowel and cervical screening uptake among Muslim women. These interventions included educational presentations, videos and information sessions that integrated religious messaging, developed in partnership with Islamic communities and religious leaders [19, 20, 24, 25, 26]. Such messaging often addressed concerns around modesty during screening [19, 20, 25], as well as emphasising the importance of disease prevention and its alignment with Islamic teachings [19, 20, 24, 25]. Although not necessarily implemented in religious settings, some faith‐based interventions are delivered in mosques [19, 24, 27].

Faith‐based interventions that included faith messaging to promote cancer screening among ethnic minorities from Christian backgrounds were also found to be effective at improving knowledge, attitudes, screening uptake and screening intentions [28, 29]. Most interventions included group education sessions alongside printed educational material. It was found that the success of such interventions was enhanced when different structural and cultural barriers were addressed in the interventions and when people from the community worked in partnership with the research team to deliver cancer education [28, 29].

### 1.1 The Intervention Workshop

In 2020, the team worked with 10 Scottish Muslim women to co‐design a culturally tailored intervention that addresses the specific barriers to screening in this population group. The co‐designed intervention is a 2‐h workshop comprising of four parts: (1) a discussion of barriers and facilitators, led by peer educators, (2) a healthcare provider‐led health education session on cancer screening and what to expect, (3) video testimonials by Muslim women talking about their experience with cancer and screening, and (4) an Islamic perspective on cancer screening presented by an Alimah (a female religious scholar) [30, 31]. The intervention was initially developed and qualitatively evaluated during a pilot phase in Scotland, which focused on its acceptability and perceived relevance when delivered online, as described elsewhere [30, 31]. The study was subsequently funded as a feasibility trial, enabling delivery of the refined intervention to a larger and more diverse sample in Scotland as well as North East England, and across both online and face‐to‐face formats.

This paper presents findings from the qualitative process evaluation conducted as part of the feasibility trial. We report participants' perspectives on the acceptability and implementation of the intervention, including the different modes of delivery, while also contributing to the feasibility assessment of a future definitive trial by examining communication and recruitment processes, as well as data collection.

## 2 Methods

As part of the feasibility trial, qualitative and quantitative data collection was conducted. For the quantitative component, before each workshop, women were asked to complete surveys, either online or on paper, asking about their knowledge of cancer and screening, attitudes towards screening and barriers and facilitators to screening (a total of 27 questions) [32, 33]. The surveys also included the religious health fatalism measure for Muslim populations (8 questions) [34] and the modesty measure for diverse Muslim populations (10 questions) [35]. After the workshops, participants were asked again to answer questions on knowledge, attitudes and barriers and facilitators to cancer screening. The survey was piloted with members of the public involvement and community engagement (PICE) group to assess the length and appropriateness of the survey tool. The survey took 20–30 min to complete. Findings from the quantitative data collection are described elsewhere (under publication).

After each workshop, participants were invited to take part in focus groups or interviews with a research team member. Participants had the option to attend an online, telephone, or in‐person interview or focus group.

### 2.1 Preliminary Steps

The community recruitment leads (FH and CS) recruited 10 Muslim women to join the PICE group. The PICE group members were of South Asian background and were active in their community; one member was involved in the initial intervention development. After being introduced to the study and their roles, the PICE group joined six meetings per year to advise the research team on intervention material, recruitment methods, data collection tools, and data analysis results. Nine PICE group members also agreed to become peer educators (total number = 15) and received four 2‐h training sessions to prepare them to help with intervention delivery.

The intervention content and delivery methods were previously co‐designed and pilot tested with Muslim women in Scotland, who deemed the workshop to be acceptable [30, 31]. In the current study, the PICE group further reviewed the intervention content and gave feedback. The intervention was planned accordingly. The survey questions were derived from validated tools. The questions on knowledge and attitudes and barriers and facilitators were taken from Cancer Research UK's Cancer Awareness Measures [32]. The religious fatalism questionnaire and Modesty measure were developed and validated with American Muslim women [34, 35].

### 2.2 Intervention Setting and Delivery

The study is set in two socioeconomically deprived areas with poor cancer outcomes: urban areas of the North‐East of England (Mainly Tyne and Wear and Teesside areas) and Scotland (Greater Glasgow area), where the mortality risk from cancer is higher when compared to the rest of the UK [36, 37, 38, 39]. Muslims only form 2.2% of the Scottish population, while only 4.2% of North East population describe themselves as non‐Christian [8]. Further, research shows that ethnic minority populations living in these areas experience worse health outcomes [37, 38, 39]. Currently available data on screening uptake does not record religion. Muslim communities living in these areas are not widely included in UK cancer screening research. One study by Campbell et al. [11] showed that Muslims in Scotland have lower uptake of bowel screening compared to the rest of the population.

The intervention was delivered 16 times, through six in‐person and 10 online workshops, with 261 Muslim women between February and June 2024 in North East England and Scotland. Following the workshops, women were invited to take part in focus groups or interviews. We conducted a total of five focus groups, three dyadic interviews and seven individual interviews.

### 2.3 Sample and Recruitment

Muslim women aged 25–74 years living in either North East England or Scotland and who were not up‐to‐date with all recommended screening (i.e. did not attend one or more screenings within the timeframes specified by the UK national screening committee for each type of screening) were eligible to participate in the intervention workshop. Eligibility was assessed through eligibility questions that asked about age, place of residence and the last time they attended breast, bowel and cervical screening.

Convenience sampling was used to recruit participants. Two recruitment leads, FH and CS, engaged with the community and placed advertisements for the study in mosques, local community centres, social media and different community locations. Interested participants contacted the research team and were assessed for study eligibility. Snowball sampling was also used through participants who attended the workshops.

Before registering for the study, all participants were given a participant information sheet (supplementary file 1) and were asked to sign an informed consent form (supplementary file 2). Participants were reimbursed for their time with £25 gift vouchers.

### 2.4 Data Collection— Interviews and Focus Groups

Participants were offered the choice to take part in either an interview or a focus group following each workshop. Data collection took place between February 2024 and January 2025. As a result, some interviews were conducted more than 6 months after workshop attendance. A topic guide (supplementary file 3) was developed based on the Theoretical Framework of Acceptability (TFA). The TFA and its seven components (Table 1), assesses whether intervention implementation or delivery is considered appropriate by those delivering or taking part in the intervention [40]. The topic guide was prepared with a semi‐structured approach, allowing participants to elaborate on issues they considered important. We continued to conduct interviews and focus groups until data saturation was reached and no further themes could be derived from the data.

**Table 1:** TFA components used to guide the qualitative assessment (40).

TFA component	Definition
Affective attitude	How participants felt about the intervention overall
Burden	How participants felt about what was required of them to take part in the workshop
Perceived effectiveness	Whether and how the intervention was perceived to change behaviour or attitudes
Coherence	Understanding of how the different parts of the intervention work to achieve the intended outcomes
Opportunity costs	Whether taking part in the intervention occurred at the expense of other commitments or required any costs
Self‐efficacy	Confidence to perform the required tasks to attend the intervention and attain information during participation
Ethicality	The appropriateness of the intervention according to the participants' beliefs and values.

Due to practical reasons and to encourage participants to take part in the qualitative evaluation, participants were given a chance to join focus groups or interviews, depending on their preference. It is understood that individual interviews can give participants a chance to elaborate on their own views and ideas, while focus groups take advantage of the group dynamic to construct ideas [41, 42]. Therefore, during focus groups, all participants were encouraged to share their views and express opinions that are different to those of the rest of the group. Participants were probed to explain their views in details [41, 42]. All study participants were drawn from the same population and all qualitative data collection methods addressed the same research questions and used the same topic guide. All data were analysed using the same method and theoretical framework.

As several participants felt more comfortable speaking languages other than English, Urdu‐English interpreters supported the focus groups, alongside a bilingual PICE member. A bilingual researcher conducted interviews with Arabic‐speaking participants and checked the translated transcripts. These measures supported the rigour of the interpretation and translation processes and helped to ensure that participants' intended meanings were preserved as accurately as possible.

### 2.5 Data Analysis

All interviews were audio recorded. Recordings were transcribed verbatim, and translated where necessary. Field notes were taken to account for the interactions between the interviewer and participant and dynamics of the group (in focus groups). Analysis started with data familiarisation through reading transcripts. Transcripts were then uploaded to NVivo 14 for coding and analysis. Two researchers, RJ and FC, independently coded and analysed the transcripts using the Framework Analysis method developed by Ritchie and Spencer [43], guided by the TFA. Coding by the two researchers was compared, and discrepancies were resolved by discussion. Codes were refined by combining those with similar meanings, and splitting those that captured different concepts. Although, the TFA's component informed analysis, inductive coding was used to develop sub‐themes, which were then mapped onto the predetermined main themes (Table 1) [44]. Data from individual, dyadic and focus group interviews were analysed as a single dataset, which required the researchers to look for similarities and inconsistencies in ideas or concepts generated from different interview methods.

The PICE groups were given a sample of the transcripts, and they were asked to identify themes or concepts in each transcript. Their views were then discussed to determine whether their interpretations were different to those by the research team, which ensured that researcher bias was minimised.

## 3 Results

Thirty‐six women were interviewed: five focus groups, three dyadic interviews and seven individual interviews were carried out. Participant characteristics are presented in Table 2. An Urdu interpreter helped in one focus group; two interviews were conducted in Arabic and translated after transcribing.

**Table 2:** Characteristics of qualitative study participants.

Demographic characteristics	Number of participants
Interview method:	
*Focus groups:* Online: In‐person:	*23 (5 focus groups)* 815
Individual interview:	7
Dyadic interview:	6
Participant location	
Scotland	24
North East England	12
Workshop mode of delivery	
Online	15
In‐person	21
*Ethnicity*	
Pakistani	21
Bangladeshi	3
Arab	5
Indian	1
Mixed	4
Other	1
Language spoken at home	
English	13
Arabic	6
Urdu	13
Bengali	2
Punjabi	1
Other	1
Education level	
Degree or higher degree	18
High school education	4
No formal qualifications	7
Prefer not to say	4
Higher education qualification below degree level	3
Age group	
25–29 years	3
30–39 years	12
40–49 years	8
50–59	2
60 and over	11

*Note:* Data are presented according to the seven components of the TFA: (1) Affective attitude, (2) burden, (3) perceived effectiveness, (4) coherence, (5) opportunity costs, (6) self‐efficacy and (7) ethicality.

### 3.1 Affective Attitude

#### 3.1.1 Delivery

All participants reflected positively on the workshop's atmosphere and how information was delivered. Familiar settings and a welcoming tone contributed to a relaxed environment.

I felt the environment was relaxing because I know the venue, I mean I don't go that much but I have been there in the past quite a lot so it's familiar and relaxing. And I felt all the various speakers they were… I don't know, they made you feel welcome, there was a warmth.

Scotland, age > 60

In in‐person workshops, women enjoyed connecting with other women from their community, either during the discussion or during lunch at the end of the workshop.

We're comfortable with this group…… Yeah. I think it helped us that way to have this talk in the group rather than going somewhere else when you've got other people there.

Scotland, age > 60

However, some women indicated concerns around privacy and feeling uncomfortable talking about cancer screening with women from their local community, which limited open discussions.

…. And I think one of the issues in our community is, “That person might know my mother‐in‐law,” or, “That person might know my mum.” Did I speak openly? Probably not because I was a little bit worried that I don't want private information going back.

Scotland, age 40‐49

Workshops led by women from the community (peer educators) were described as enjoyable and accessible, facilitating sharing and communication in one's own language.

We were divided in groups that people could speak their local language and share.

NE, age 30‐39

In contrast, women attending online workshops often described difficulties connecting with other participants due to the online format.

It is a bit scary because there are quite a lot of people that you don't know and you're not seeing face to face, but it was okay.

Scotland, age 30‐39

Most appreciated the language and the methods used by the GP to explain cancer screening, which they found clear and accessible.

It wasn't‐ the jargon used was jargon friendly for us. Medically we didn't want to hear big words and, yeah, I think it was really good, it was planned…

Scotland, age > 60

But you gave me the information in a different way, for example, by presentation, by a graph, so it is very nice, and easy for the learner to gain the information easily.

NE, age 40‐49

Language accessibility was identified as a strength, whilst having translations and interpreters present in the workshop was highlighted by most as important for women who were not fluent in English; some women suggested the workshop could be delivered directly in the participants' native languages.

‘Speaking Urdu, rather than being translated, is direct and they have a direct connection and they have a direct kind of response to what you're saying. And time‐saving.

Scotland, age 40‐49

As workshop materials were mostly in English with interpreters in the room, some participants suggested that translating the videos or slides into various languages could further enhance their effectiveness.

If possible, we could add the video in other languages. Some people watched it and didn't understand it. It was a feeling they got from her… We have a diverse culture. It could have been translated.

NE, age 30‐39

#### 3.1.2 Content

Participants said that the different workshop components were helpful, and that the workshop delivery accommodated different needs.

There was a group of people who covered everything. There was a translator, a video, a slide, a room for discussion, a small group. There were answers to all the questions. There was information for all the screenings. I don't know what else to add.

NE, age 30‐39

A few participants also described that having videos of Muslim women talking about their experience with cancer and its screening gave a *‘human touch’* to the workshop, which made the topic relatable and come alive.

I think listening to them and hearing their perspective hits home a lot better and makes you think that this is the impact it can have on your family so you need to get yourself checked.

NE, age 40‐49

A few women indicated they would have also liked to listen to other perspectives from Muslim women, such as the experience of younger women, the views of women who have never been to screening or the experiences of a cancer survivor. They emphasised that having multiple stories could be more relatable or impactful. Having women talk about their experience in‐person would allow them to have a chat with the speakers afterwards.

Well, you could have had the real woman, I think, maybe talking about it, and it probably would have stayed with me longer. Or one or two real women, you know, speaking about it.

Scotland, age 40‐49

### 3.2 Burden and Opportunity Cost

#### 3.2.1 Location

Most participants described attending the workshop as convenient and requiring minimal planning. In‐person venues were perceived as convenient and accessible to most.

The place you chose is also appropriate. This place is close to our homes, so it's easy to reach. It's not a far place. If it's a far place, you won't see this number of people attending. It will be half or a quarter. Honestly, the place is nice.

NE, age 50‐59

When workshops were delivered with existing community groups, participants described that there was no additional effort on their part to attend the workshop, as they are usually present at the venue at the scheduled time of the workshop. However, a few participants suggested that they would have preferred a different location and that some workshop venues could be improved by better parking and public transport links.

You could maybe make it more central, rather than quite far out over there… So you could probably make it where there's a bigger car‐parking facility, and that would make it more accessible to public transport as well.

Scotland, age > 60

Most participants who attended in‐person workshops indicated they were satisfied with the venue and workshop setup, highlighting the comfortable and sociable environment.

Well, you provided information, you provided a comfortable environment, you provided people to meet in a nice place. And you made it sociable, by providing coffee and tea, so they don't have to just sit around and be forced to listen.

Scotland, age 40‐49

A few participants noted that communication about the workshop schedule could have been clearer beforehand.

I just tend to think if I plan things and I rush them then I get a bit flustered so that's what I… I made sure that I went early……

Scotland, age > 60

Online delivery offered additional convenience, particularly for mothers of young children.

For me, [online is] easier…. Easier than going by bus, or… I don't have any problem. Some people complain about [having to do things online in] 2020. No, me, I don't have any problem….

NE, age 40‐49

I guess I liked that it was online because I've got a son, so it's easier than having to go in person because then you have to get them looked after by someone.

Scotland, age 30‐39

Despite this convenience of online delivery, a few mentioned technical difficulties when accessing the workshop.

It was a bit challenging for me, a little bit challenging because of network issues, but it was okay in the end, overall it was okay.

NE, age 50‐59

#### 3.2.2 Organisation

For both online and in‐person workshops, women felt that they were well‐organised, with clear structure and delivery.

Zoom was okay. The camera was clear. The slides that you put up. There was a translation in Arabic and in Urdu for the people. You divided us into groups. Everyone had a group leader. I don't have anything negative, to be honest.

NE, age 25‐29

#### 3.2.3 Timing of Workshops

In‐person workshops were delivered mid‐morning and online workshops were offered at a range of times, including daytime and evening sessions. Participants indicated that timings were suitable and could easily be integrated in their day, especially for mid‐morning sessions.

The time was appropriate. It's the time for children to be in schools. We can come and attend such events.

NE, age 50‐59

Evening online workshops were particularly helpful for working women, who otherwise would not be able to attend, and allowed them to balance workshop attendance with family responsibilities.

The good thing is that the time was appropriate. It was at 7 or 8. I had dinner with my children. I changed their clothes. I did the homework with them. The time was excellent….

NE, age 30‐39

While some participants initially perceived the 2‐h duration as long, most found the length acceptable when considering the content and amount of information delivered. Additionally, a few described that the workshop was engaging and enjoyable, and therefore, the 2 h did not feel too long. Delivering the content in a single workshop also reduced the burden for some women, it required them to attend only one session instead of multiple sessions, which might have been difficult for them to manage.

I thought, “Oh, it's going to be too much. Who is going to sit for 2 h?” But when I sat down, and when the workshop started, I didn't even feel it. I'd just made a cup of tea, and I hadn't even finished it and the workshop finished, and I thought, “Oh my God, 2 h has gone so fast.”…….

NE, age 25‐29

However a few, mostly older women, mentioned that concentrating for 2 h could be difficult. Having a break during the workshop was helpful for some.

A break is good to have, it takes you off from the intensity of the subject, and lets people chat. Because one of the things, when you bring people together, is for them to talk to each other about the subjects you've raised. So it's useful.

Scotland, age > 60

In contrast, others said they would opt out of having a break as it could disrupt their concentration and extent the overall workshop duration. Some women suggested that the workshop could have included more interactive activities, such as games, to increase engagement.

A game should be played to make the workshop a little bit lively……. So I think in the next workshop, there should be a game provided, like a little bit of a game like pass the mic….

Scotland, age 40‐49

#### 3.2.4 Surveys

Although the duration of the workshop itself was acceptable for most participants, completing surveys before and after the workshops was commonly perceived as burdensome.

It was quite long because you had the survey at the beginning, then the discussion, then the presentations, then the question and answers at the end.

Scotland, age > 30‐39

Some participants reported reduced engagement with the survey due to its length.

And to be honest with you, personally, I'm not lying to you, when it was the first survey, and I was doing it, I did a few questions, I read it properly…. and then after that, I was reading, like, two or three words, and I was just clicking any option…

Scotland, age 30‐39

Some talked about the importance of the pre‐ and post‐workshop surveys to assess what they learned in the workshops, and were happy to complete them despite inconvenience.

### 3.3 Coherence

#### 3.3.1 Understanding the Goal of the Workshop

All participants reported an increased understanding of the importance of cancer screening and early detection following the workshop. They recognised that finding cancer at an early stage can improve treatment outcomes and prevent disease progression. Participants also highlighted that combining Islamic perspectives and medical information worked well and this element should encourage more women to go for screening.

Although you might have that information and awareness and you might have a GP, but having that Islamic touch, it just kind of pushes it over the edge, like, ” “Yeah, this is important. From an Islamic point of view, we should be getting this done as well.

Scotland, age 30‐39

#### 3.3.2 Order of Workshop Components

When reflecting on the order of the different parts of the workshop, most described that the order was appropriate and made sense to them, particularly placing the Islamic perspective after the GP's presentation, which helped contextualise the medical information.

It was also good that you had it [the Alimah's presentation] after the GP's presentation. So you gave us all the information about screening and the Alimah then linked it to Islam. I thinks it's good that you did it this way not the other way around.

NE, age 30‐39

Only one participant felt that the *Alimah*'s talk would have been more helpful at the beginning to help engagement.

Maybe having that [the Alimah's presentation] near the beginning, if it can fit in, just because I feel like then people… like, not for myself but if other people get that Islamic perspective right at the beginning, they might be more tuned in to the other things as well.

Scotland, age 30‐39

### 3.4 Ethicality

#### 3.4.1 Suitability of the Workshop's Environment

All participants expressed feeling comfortable when attending the workshop. They especially appreciated the ‘female‐only’ setting, which enabled open discussion of sensitive topics.

And the good thing is the privacy, it's only women, no men, so you can talk properly, openly, and you know what we were saying, it's going to be just staying here, and not going anywhere.

NE, age 25‐29

Some participants discussed that online delivery also supported privacy as there was the option to keep the camera off.

I liked that you could maybe have your video on or off because some people might not want to.

Scotland, age 30‐39

#### 3.4.2 Alignment of Workshop Content With Beliefs

Participants consistently shared they valued the message about how Islam encourages women to go for cancer screening and that they are encouraged to take care of their own health. Faith messaging reinforced the importance of screening and acted as an enabler.

Yeah, definitely, because if they see that as something that's part of their religion it's obviously going to be a higher motivator for them…

NE, age 40‐49

Participants also distinguished between culture and religion, and agreed that cultural misconceptions rather than Islamic teachings could prevent women from attending screening. A few participants described how some Muslims think that their belief in fate and that everything is written by Allah, is a barrier to health seeking but they appreciated that the *Alimah* addressed these fatalistic beliefs by recommending to get treatment when ill.

Participants highlighted that the Alimah helped clarify some of their questions about how screening would fit into their Islamic practice.

It helped clarify a lot of things for us. For example, things like doing a smear test and Ramadan. She explained that it wouldn't break our fast, which is something we didn't know before.

NE, age 30‐39

All participants expressed that cancer and screening are rarely discussed within their communities. They also expressed that a stigma is associated with cancer, as some people kept it as a secret. Therefore, they felt raising awareness is necessary.

Because a lot of it is because of culture and people feeling stigma about doing it. So this was helpful for women to know.

NE, age 30‐39

There are people who don't have any background in this matter. So, it's good that you made it specific to Muslim women.

NE, age 30‐39

#### 3.4.3 Appropriateness of Data Collection Methods

Although most women indicated that the survey was long, overall they found the survey questions appropriate. However, some women, specifically from one community centre in Scotland, found the religious questionnaires culturally inappropriate. They interpreted the questions as implying that Muslim women do not seek medical care because of their religion. Women described that their Islamic beliefs do not stop them from taking care of their health.

So, that [religious questions] was very off‐putting, because that made out that most [ethnic minority] or Muslim women don't go for screening or don't go for treatment, because they feel that it's given by God, so, you know, you have to bear it.

Scotland, age > 60

### 3.5 Self‐Efficacy

#### 3.5.1 Ability to Engage in the Workshop

Whilst the workshop environment was generally supportive, some participants noted that the interactive element of the discussions could feel uncomfortable for those who were shy or preferred to listen rather than contribute.

The only thing, I know some of them were a little bit uncomfortable when they went into workshops and they had to talk a little bit. That was something that some of them weren't very keen on because they were shy and they got embarrassed, and they prefer to listen.

NE, age 40‐49

#### 3.5.2 Ability to Engage in Screening

Participants shared that the workshop content and information presented by the GP and *Alimah* increased their knowledge of screening, and motivated them to go for screening. Practical advice and clear information provided by the GP about screening improved understanding and reduced perceived barriers.

I think the recommendation by the GP was quite good of how to make it [bowel screening] less messy. That was quite good.

Scotland, age > 60

Addressing concerns about modesty and fears about exposing their bodies to male health professionals were recognised as barriers to some Muslim women. Therefore, the advice about the ability to request a female professional motivated them and was found especially helpful.

### 3.6 Perceived Effectiveness

#### 3.6.1 Trusted Sources of Information

Most participants valued information delivered by credible sources and described that they were reassured that the workshop information was presented by healthcare professionals’. It's good coming from a medical point of view as well. It's not just a person telling you about it. It's a doctor. “You feel more comfortable, and then you believe in it more as well”.

Scotland, age > 60

Although all participants appreciated that a GP was present at the workshop, some said that they would have preferred having a cancer specialist.

Well you might, I suppose, bring in actually a specialist rather than a generalist, who can answer more in‐depth questions and is more confident about what they're speaking about.

Scotland, age 40‐49

#### 3.6.2 Changed Attitudes Towards Screening

Most participants described changes in attitude towards screening following the workshop. Some mentioned that before the workshop they missed screening invites; a few highlighted hesitancy to go to cervical screening or to return the bowel screening kit. After attending the workshop, they either requested appointments, responded to invitations they had received, or expressed they would go to screening when they received the next screening invite.

I had a fear of going for the test for her breast screening, but when I came to the workshop, I felt more comfortable and felt more aware and the reasons why I should go. I went for my breast screening and I'm okay about it.

Scotland, age > 60

It encouraged me to go to smear test. I went to my smear test for the first time after the workshop. I would have never done that before the workshop.

NE, age 40‐49

Participants also revealed feeling encouraged to discuss cancer and screening with their family and friends, and possibly encourage others to go to screening.

I could go and tell my mum and dad about that because they were over 50, and then when I asked mum, she was like, ‘Yeah, the GP sent me this but I've not sent it back’, and I was like, ‘Oh, you should get that sent in now’. (Laughter) So, yeah, that was good.

Scotland, age 30‐39

Although most believed that the workshop positively influenced women's attitudes towards screening, they asserted that some women might still experience stigma in relation to cancer screening and feel embarrassed talking about it, which might stop them from going to screening.

Some women wouldn't go no matter what you tell them, so I don't think there's anything else we could do about it.

NE, age 30‐39

A few also mentioned that although having a workshop for Muslim women was helpful, including younger women and men would be important too.

I know a lot of young girls, younger than 25, who had breast cancer at a very young age. We knew that breast cancer affects older women who had a lot of children or have other conditions…… So we shouldn't only educate older women. We should also educate younger women.

NE, age 30‐39

## 4 Discussion

This qualitative component of the feasibility study explored the acceptability of a peer‐led, faith‐based intervention to improve breast, bowel and cervical screening uptake among Muslim women in Scotland and North East England in the context of a feasibility trial. Building on our earlier work, this study draws on a larger and more diverse sample, includes both in‐person and online intervention delivery, and provides insights into feasibility aspects for a future definitive trial.

Participants described learning new information about cancer screening in a relaxed and supportive environment. The integration of Islamic perspectives with medical information was consistently highlighted as valuable, reinforcing the importance of delivering health information relevant to participants. Participants reported more positive attitudes towards cancer screening following the workshop, with some reporting actually participating in screening. These findings show the importance of such community interventions and the issues that need consideration for future implementation.

There are several barriers that can prevent Muslim populations from attending screening. Research on screening attendance conducted in the UK, the United States and Middle Eastern countries, along with findings from our preliminary work, show a complex web of barriers related to the health system, knowledge and awareness, and cultural beliefs [30, 45, 46, 47, 48]. Familiarity with the health system and how to access screening was cited as a barrier among migrant Muslim populations in South East London [46]. Knowledge and awareness of breast, bowel and cervical cancer and screening, such as knowledge of eligibility for screening, can also be a hindrance [45, 46]. Cultural factors, include concerns regarding modesty, the role of women in the family and fatalistic beliefs contribute to low uptake of cancer screening [45, 46, 47, 48]. Feedback from our qualitative interviews shows that our intervention addressed a range of these barriers to cancer screening faced by Muslim women, including lack of knowledge about the screening process and importance of early detection, Islamic perspectives on screening and the ability to maintain modesty during screening.

Participants valued the workshop content and reported learning new information about cancer screening. Limited understanding of screening has previously been found as a barrier to breast and cervical screening among Muslim women [15, 45, 46]. Similarly, lack of knowledge regarding how to perform the bowel screening test might deter some and may contribute to the ‘disgust’ factor [49]. In this study, the GP's explanation of how to complete the FIT test with provided practical tips appeared to help women overcome this barrier. Raising awareness of cancer screening has been found to improve uptake among Muslim communities [24]. In this study, the varied workshop activities and presentations appeared to engage participants, who expressed that the video and visuals included in the presentations helped retain information. Integrating visuals and videos in education interventions was also found in other studies to enhance comprehension and information attainment compared to traditional didactic methods [50, 51]. Having a Muslim woman share her personal experience of cancer screening positively influenced participants. Listening to a peer's story can promote learning and enhance engagement, and inspire a change in attitudes and behaviour [52, 53, 54]. However, a few preferred having a woman talking about her experience in‐person rather than on a video, which might be beneficial although not always feasible, which from a feasibility and scalability perspective is important to consider. First‐person storytelling, either by video or in‐person, can have a positive effect on people's health behaviours and attitudes. However, it is not clear whether in‐person storytelling can offer additional advantage [55].

Delivering a workshop specifically for Muslim women, and led by Muslim women, was viewed positively by participants. This created a safe and relatable space where most women felt able to talk freely and build connections. Partnering with communities to deliver health awareness interventions can facilitate knowledge exchange and strengthen engagement, with potential benefits extending to the wider community [56].

Participants valued the use of jargon‐free language in the workshops, while the availability of interpretation facilitated engagement among non‐English speakers. This is particularly important as language barriers and low health literacy are known barriers for Muslim women to accessing screening services [15, 17, 57]. Accessible communication, including using plain language and translated health education materials, is therefore essential to improve cancer screening uptake [58, 59, 60]. However, much of the health information currently available in the UK is not sufficiently tailored to meet these needs [61].

The study also worked to include migrant populations who have lower English proficiency. Migrant populations often express multiple barriers to accessing health services due to language barriers and low knowledge of the health system and how to access it [62, 63, 64]. Having basic English language skills does not necessarily mean that migrants are health literate and can discuss health information, especially when specialist language is used [62]. Further, migrant communities are included in health research less often than the rest of the population due to several factors, namely language barriers, trust in researchers and lack of outreach from research teams [62]. Involving the community in research design and delivery can ensure that the research is appropriate and meets the needs of the community [65]. Reaching out to ethnic and migrant communities through trusted community members can enhance their participation in research [62]. The IMCAN Study worked to meet the needs of the UK Muslim population, including the migrant population, through a participatory approach where Muslim women were involved in intervention design and delivery which ensured that the intervention was acceptable. Further, recruitment was led by two trusted members of the Muslim community which ensured trust.

Having information about cancer and screening presented by a GP reassured participants that the information presented was credible and accurate. Due to the overwhelming amount of medical information available online, individuals often seek to verify the information with their GPs, but this is not always possible due to difficulties getting timely appointments [66, 67].

Participants' views on workshop duration varied. While some felt that the two‐hour duration was appropriate, others reported difficulty concentrating for this length of time. Similarly, short breaks seemed helpful to some participants, allowing them to refresh and re‐engage, but this was not a common view for all. This suggests there is no single optimal workshop duration that suits all participants. However, having some flexibility in delivery and incorporating short breaks could be helpful since this can help regain focus and enhance learning [68]. Although the workshop duration was suitable for most, the need to fill in surveys before and after the workshops added to participant burden; some expressed that the surveys were lengthy. In future studies, the research team, with the help of PICE members, will work to refine survey questions based on the feedback received by participants. In the current study, the survey was administered online and on paper, with help from interpreters and the research team when needed. In the future, the survey format will be revised to ensure that participants can fill in their answers more easily.

The Islamic perspective on cancer screening, presented by the *Alimah*, was new to participants and consistently valued by participants. Participants shared that cultural misconceptions, often confused with Islamic teachings, may discourage women from prioritising their health. Previous studies have similarly reported that stigma can limit discussions about cancer or cancer screening within families or community [45, 46, 48]. Beliefs around prioritising family's health before their own or viewing disease as predetermined by Allah may act as barriers to participating in screening [45, 46, 47, 48]. In contrast, the messages presented by the *Alimah* in this study resonated with participants, as they reflected Islamic teachings in the Quran, which advise Muslims to take care of their own health and seek medical care when needed [69]. This suggests that faith‐based messaging, when grounded in accurate religious interpretations, functions as an enabler and can challenge misconceptions and encourage engagement with screening.

Similarly, modesty is important for Muslim women, and the concept stems from Islamic teachings. It has been found that concerns about maintaining modesty can discourage Muslim women from attending breast and cervical screening [70, 71]. In this study, the explanation provided by the *Alimah* about the ability to request that a woman do the screening, and that Islam allows having a man to perform medical procedures if necessary, could have helped Muslim women overcome barriers to screening.

Interventions that utilise a faith‐based approach appear acceptable and show potential to change attitudes towards cancer screening. Acceptability of religiously‐tailored workshops to improve cancer screening uptake among Muslim populations was explored in previous studies conducted in the United States; participants in these studies also appreciated the culturally and linguistically tailored content and delivery [20, 25, 72]. Acceptability was further increased when community members and religious leaders were involved in intervention delivery [20, 72]. Evidence on the acceptability of culturally‐tailored interventions to improve cancer screening among British Muslims remains limited. This qualitative paper builds on our earlier pilot work and contributes to addressing this gap, with findings that support the implementation of such culturally and faith‐informed interventions to improve cancer screening uptake among Muslims in the UK context.

In addition to acceptability, participants identified several aspects of the intervention that may have implications for feasibility in a future trial, particularly in relation to survey burden and communication. A more detailed assessment of trial feasibility will be reported separately.

### 4.1 Strengths and Limitations

This study builds on our earlier pilot work, which included an initial qualitative evaluation of a co‐designed, faith‐based intervention. It extends this work by drawing on a larger and more diverse sample, incorporating both in‐person and online delivery, and exploring acceptability with the context of a feasibility trial. In contrast to the earlier evaluation, the use of the TFA strengthens the methodological rigour of the analysis, enabling a more systematic and theory‐driven exploration of participants' perspectives. Findings from this qualitative evaluation demonstrate the intervention's acceptability and its potential for changing attitudes towards cancer screening.

However, these qualitative findings might not be generalisable to the wider population. First, the study was conducted in two locations, North East England and Scotland, which may not be representative of the entire Muslim population in the UK. Second, the use of convenience sampling may have resulted in the inclusion of participants who already had positive attitudes towards cancer screening. In addition, self‐selecting into the qualitative component interviews/focus groups may have led to an overrepresentation of women who had a positive experience in the workshop. The use of different qualitative data collection methods could have resulted in inconsistencies in data generated. To overcome this, the researchers made notes on the interactions between interview participants and looked for any inconsistencies in the codes generated by each method. Here, it was found that the data from individual interviews confirmed data from focus groups, which enhanced understanding of participants' experience with the intervention.

Despite these limitations, the evaluation included 36 Muslim women from diverse backgrounds and were encouraged to speak freely and reflect on any aspects that they think need improving. Difficulties in recruiting women for the qualitative evaluation caused delays in data collection, with some interviews and focus groups conducted up to 6 months after workshop attendance, which may have introduced recall bias.

## 5 Conclusion

Using culturally tailored peer‐led, faith‐based interventions to promote cancer screening in Muslim communities shows promise in improving knowledge of, attitudes towards and uptake of screening. Recognising the diversity within communities and providing information that is accessible and culturally safe and relevant improved women's experiences of the intervention. Using interpreters further supported women who are not fluent in English. Findings from this evaluation indicate high levels of acceptability; however, these need to be supported by quantitative analyses of changes in knowledge, attitudes and behaviour. Overall, findings indicate that this intervention offers a promising approach to encouraging cancer screening uptake among Muslim women.

## Author Contributions

**Rawand Jarrar:** writing – original draft, investigation, project administration, formal analysis, writing – review and editing, methodology, data curation. **Kathryn A. Robb:** conceptualisation, investigation, funding acquisition, writing – review and editing, methodology, project administration, supervision, resources. **Marie K. Murphy:** conceptualisation, investigation, funding acquisition, writing – review and editing, methodology, project administration, supervision, resources. **Cerysh Sadiq:** investigation, methodology, writing – review and editing, project administration. **Anna Matthews:** investigation, writing – review and editing, methodology. **Fozia Haider:** investigation, methodology, writing – review and editing, project administration. **Alex McConnachie:** conceptualisation, investigation, funding acquisition, writing – review and editing, methodology, supervision. **Aasim I. Padela:** conceptualisation, investigation, funding acquisition, writing – review and editing, supervision. **Floor Christie‐de Jong:** conceptualisation, investigation, funding acquisition, writing – original draft, writing – review and editing, methodology, project administration, data curation, supervision, resources.

## Ethics Statement

The study was approved by the University of Sunderland Research Ethics Committee (Reference Number 020011) and the NHS Health Research Authority (reference24/LO/0179). Before taking part in the study, all participants provided informed consent. Each participant received a £25 gift voucher after the interview or focus group.

## Conflicts of Interest

The authors declare no conflicts of interest.

## Data Availability Statement

Anonymised study data may be shared with other researchers in line with participant consent. Data are not publicly available but may be accessed upon reasonable request, subject to appropriate data sharing agreements and ethical approvals.

## Supporting information


Supporting File 1

Supporting File 2

Supporting File 3
