# Supplementary material for: Acceptability of a Peer‐Led Faith‐Based Intervention to Improve Breast, Bowel and Cervical Cancer Screening Uptake Among UK Muslim Women: A Qualitative Evaluation
Source: Health Expect. 2026 Oct 16;29(5):e70935. doi: 10.1111/hex.70935 (PMC13674415; doi:10.1111/hex.70935)
Supplement: Supplementary file 1 [file HEX-29-e70935-s001.docx]

Improving Muslim Women Cancer Screening Uptake (IMCAN study)

Participant consent form

We are asking if you would like to take part in the above research project. Before you consent to participating, we ask that you please read the participant information sheet. If you have any questions or queries before completing the consent form, please speak to the principal investigator, Dr Floor Christie-de Jong ([floor.christie@sunderland.ac.uk](mailto:floor.christie@sunderland.ac.uk)) or Research Associate Dr Rawand Jarrar ([rawand.jarrar@sunderland.ac.uk](mailto:rawand.jarrar@sunderland.ac.uk))

1. I confirm that I have read the participant information sheet and fully understand what is expected of me within this study
   - Yes
   - No
2. I confirm that I have had the opportunity to ask any questions and to have them answered.
   - Yes
   - No
3. I understand that my participation is voluntary and that I am free to withdraw at any time without giving any reason, without any adverse consequences or penalty.
   - Yes
   - No
4. I understand that if I withdraw from the study, my data collected up to that point will be retained and used for the remainder of the study.
   - Yes
   - No

5. I understand that I will be participating in a 2-hour workshop, and I am aware of the main parts of the workshop.
   - Yes
   - No
6. I understand I will be asked to fill in questionnaires collected at the beginning of the study, 6 months and 12 months after the workshop.
   - Yes
   - No
7. I understand that I will be asked to share my NHS number with the research team in order for them to look at my screening attendance. (I understand that this is voluntary, and I have the right to refuse without affecting my participation in the study).
   - Yes
   - No
8. I understand that I will be asked to take part in a group discussion after the workshop in order to share my experience and views on workshop content and delivery. (I understand that this is voluntary, and I have the right to refuse without affecting my participation in the study).
   - Yes
   - No
9. I consent to anonymised data from my questionnaire to be used in study reports and future publications.
   - Yes
   - No
10. I consent to securely storing my questionnaire answers for 3 years after the study has finished and I agree that information from the questionnaires may be passed as anonymous data to other researchers and made available for future research.
    - Yes
    - No
11. I agree to take part in the study.
    - Yes
    - No

Please type your name in the box below to indicate agreement to participate in the study.
