# Supplementary material for: Acceptability of a Peer‐Led Faith‐Based Intervention to Improve Breast, Bowel and Cervical Cancer Screening Uptake Among UK Muslim Women: A Qualitative Evaluation
Source: Health Expect. 2026 Oct 16;29(5):e70935. doi: 10.1111/hex.70935 (PMC13674415; doi:10.1111/hex.70935)
Supplement: Supplementary file 2 [file HEX-29-e70935-s002.docx]

Improving Muslim Women Cancer Screening Uptake

Participant information leaflet

# Introduction

We would really like your help with our research project. Before you decide if you want to join, please read this paper carefully. If you have any questions, you can contact the people listed at the end of this paper.

# Information about the study

## What is the main focus of this study?

The main focus of this study is to help more Muslim women in the North East of England and Scotland take part in breast, bowel, and cervical cancer screening. Cancer screening can save lives by finding cancer at an early stage. Finding cancer early is key to treating it successfully and can improve survival.

In the UK, the NHS offers regular screening and early detection for breast, bowel and cervical cancer. These three types of screening are offered for women registered with a GP. The ages for each screening programme vary (see table below).

| **Screening programme** | **Age range** |
| --- | --- |
| Breast screening | 50 to 70 years |
| Bowel screening | 60 to 74 years |
| Cervical screening | 25 to 64 years |

## Why is the research being done?

Research has found that Muslim women are not going for cancer check-ups as much as recommended. There are many reasons for this, like not knowing about the check-ups, feeling embarrassed, or being worried about seeing a male doctor. This research aims to encourage more Muslim women to get cancer screening by providing them with necessary information to make informed choices.

## Why have I been invited to take part?

We would like to invite you to take part in this research study if you are a Muslim woman living in North East England or Scotland, and you are between 25 and 74 years old and haven't had all of your recommended cancer screenings before. Your participation is valuable to us!

## Do I have to take part?

No, participation is voluntary. You can decide if you want to be part of this research or not. It's completely up to you, and there's no pressure to participate.

## What will happen if I agree to take part?

If you choose to join, you will be asked to fill an online questionnaire. Then, there will be a two-hour workshop which you can attend online or in-person. The workshop aims to give you information about what happens during screening and you’ll have a chance to ask any questions about cancer screening.

The two-hour workshop includes four parts (see figure below): 1) An informal group discussion about what makes it easier or harder to get cancer screening, 2) A session led by a female GP where you learn about cancer screening and its process, with the opportunity to ask questions, 3) Videos of Muslim women sharing their experiences with cancer screening, and 4) A talk led by a female religious scholar (Alimah) on how cancer screening is viewed in Islam, also with the opportunity to ask questions.

Figure 1 Workshop components

## What happens after the workshop?

After the workshop, you will be asked to fill another shorter questionnaire, which we will ask you to fill in again after 6 months and 12 months. You might also be asked to join a group discussion to share your thoughts about the workshop.

After the workshop, we will also ask you if it's okay to check with the NHS about your cancer screening after 12 months. This helps us to see if the workshops really encourages women to go to cancer screening. This is not compulsory and deciding not to share this information with us will not affect your participation in the study.

**Deciding to go for screening**

The research team will not refer you to the GP or make your screening appointment. If you fall in the right age group and would like to get screened, have missed an appointment, think you should have received an appointment, or a bowel screening kit, please see below who to contact:

**England**

1. For cervical cancer screening (age 25-64): please contact your GP
2. For breast cancer screening (age 50-70) : please contact your local NHS breast screening service [here](https://www.nhs.uk/conditions/breast-screening-mammogram/how-to-book-or-change-an-appointment/): https://www.nhs.uk/conditions/breast-screening-mammogram/how-to-book-or-change-an-appointment/
3. For bowel screening: please phone the national helpline: 0800 707 60 60:
   a. if you have any questions or ask for a kit if you need a new one
   b. if you’re over the age of 74and would like to receive a kit every two years

## Will I receive any payments for participation?

If you take part in the workshops, we will give you a £25 retail gift voucher as a reimbursement for your time and travel expenses. This is a Love2Shop gift voucher (<https://www.love2shop.co.uk/>). If you’re taking part in the in-person workshops, we’ll give you the voucher as a hardcopy at the end. If taking part online, the voucher will be emailed to you.

If you agree to take part in the group discussions about the workshop afterwards, we will also reimburse you for your time through another £25 voucher.

For the follow up surveys at 6 months and 12 months, we will give you a £10 voucher each time.

## Are there any risks for taking part?

Taking part in this research shouldn’t put you at risk or in any danger. But if you have any worries, please just let us know. We want you to feel safe and comfortable. If you feel stressed during the workshop, you can choose to leave the workshop. Our GP and Alimah are also available for you and will stay behind at the end of the workshop to answer any private questions you might not want to ask in front of the group. As our GP does not have access to your medical history, we recommend contacting your own GP if you have any specific worries about your health. For immediate emotional support, you can contact the Samaritans at any time by calling 116 123.

## What are the benefits of participating in this research?

You may enjoy the workshop as it may give you information about screening and may help you overcome some difficulties that may have stopped you from getting screening before. You coming to the workshops and sharing your thoughts about the workshop can help us develop a programme to make it easier for Muslim women, living in the UK, to go for breast, bowel, and cervical cancer screening. Your help can make a positive difference for Muslim women.

## Will my information be kept confidential?

Your part in this research will be kept confidential. Your personal information, answers in the questionnaire and information about cancer check-up will stay private. Only the research team can see them, and they keep everything safe on special computers with passwords. Your information won't be shared with anyone else.

## Can I change my mind about taking part?

You can decide if you want to take part or not, and you can change your mind at any time. If you decide you no longer want to participate, you can withdraw from the research. It's completely up to you, and you have the freedom to make that choice.

## What will happen to the results of the study?

The study's findings will help us create a programme to help more Muslim women in the UK get screened for cancer. We will share the results with researchers, doctors and other healthcare professionals at conferences and in scientific journals, so they can learn from it and make better decisions about healthcare.

## Who is organising and funding this research?

This research is organised by the University of Sunderland in collaboration with the University of Glasgow, and researchers from several other Universities and organisations, including Muslim organisations.

The study is funded by Cancer Research UK.

## Who has reviewed the study?

The University of Sunderland Research Ethics Group has reviewed and approved the study, as well as the Health Research Authority’s Research Ethics Committee.

## What happens next?

If you want to join the study or if you have more questions, please get in touch with the research team using the contact information provided below. They will be happy to help you.

# Contact details:

If you want to be a part of this study or if you need more information about participating, please contact the following:

Fozia Haider (Research Assistant): [Fozia.Haider@sunderland.ac.uk](mailto:Fozia.Haider@sunderland.ac.uk)

If you need further details about the research project, please contact:

Dr Rawand Jarrar (Research Associate): [Rawand.Jarrar@sunderland.ac.uk](mailto:Rawand.Jarrar@sunderland.ac.uk)

Dr Floor Christie-de Jong (Principal Investigator): [floor.christie@sunderland.ac.uk](mailto:floor.christie@sunderland.ac.uk)

Phone: 0191 5153445

Professor John Fulton (Chair of the University of Sunderland Research Ethics Group, University of Sunderland): john.fulton@sunderland.ac.uk

Phone: 0191 515 2529
