# Supplementary material for: Acceptability of a Peer‐Led Faith‐Based Intervention to Improve Breast, Bowel and Cervical Cancer Screening Uptake Among UK Muslim Women: A Qualitative Evaluation
Source: Health Expect. 2026 Oct 16;29(5):e70935. doi: 10.1111/hex.70935 (PMC13674415; doi:10.1111/hex.70935)
Supplement: Supplementary file 3 [file HEX-29-e70935-s003.docx]

**IMCAN study**

**Participants interviews and focus groups**

**Topic guide**

**Arrival**

- Find a comfortable seat
- Turn your camera on, if you can, it will be so nice for us to see your friendly faces and it makes chatting to each other so much easier. We will only record the sound for this meeting, not your cameras
- Turn your microphone off for now
- Set your screen to Gallery View, so you can see a few more of us
  - If you’re using a phone swipe left to do this
  - On an ipad or tablet tap the screen and you should see a little grid for gallery view on the left
  - And on a computer screen there’s a little grid in the top right corner that says View
- Try and open the chat window
- Find the reaction buttons and give us a thumbs up
- Type in the chat or wave at us if anything isn’t working

**Focus Groups Introduction (5 min)**

Our meeting should last about an hour or so, so we should be finished by X

The purpose of this meeting is to get your opinion of the workshop we had on [date]. Our research is trying to find out how we can encourage Muslim women to use cancer screening through their faith, because we know that health is quite important in Islam, but Muslim women seem to struggle more than women in the general population with using cancer screening services. We worked with a group of 10 Muslim women to find out what’s most difficult about cancer screening for women in Scotland and which teachings from Islam can support and encourage women to use cancer screening—and then we planned the community meeting with this group of women. So now we’re keen to know what you that meeting was like for you.

So, there’s no right or wrong answers, so don’t worry about saying a wrong thing. We want to hear all of your opinions, and also: we might ask some questions where the answer seems really obvious to you, that’s just cause we don’t want to assume things.

If you do need to leave early unexpectedly, that’s okay. Taking part in our meetings is voluntary, so you can leave at any time you want and you don’t have to answer any questions or talk about anything you don’t want. If your kids or your pets appear on the screen that is fine, too.

You might feel that the information you might share in this group are personal to you, so I want to assure you that your names will not be used in any reports of this study. But I must also ask you to respect each other’s confidentiality and that means anything said in this meeting isn’t for discussion with anyone outside this meeting. I will record the meeting when we get going, but we will only use the sound and not your pictures. (That’s why I’m wearing the big headphones, so I can hear you better.) The recording will then get typed up, so we can read through it and summarise it, so I just want to reassure your names will not be mentioned in the typed version or in any reports of our research.

We’ll start in a minute and I will tell you about some topics that I would like your views on. In principle, you can speak whenever you feel like it, but some of you may be more quiet and others may like to talk a lot, so sometimes I might interrupt you, but this is only to give others a chance to talk as well or I might call on you directly to hear your views.

Now, I think I’ve been going for long enough. Let’s do a quick round of introductions to begin.

**Topic Guide**

1. Introductions, explanations, etc. (10 min)

**TURN RECORDERS ON**

2. Experience of the intervention (15 min)

- on [date] you joined our online/in person community meeting. Thinking back, can you tell me what it was like? (affective attitude)
  - What did you think of the meeting?
  - How do you feel about the meeting now?
  - What did you like about the meeting?
    - What did you like best about the meeting?
  - If we had this meeting again is there anything we should change?
    - Is there anything we should change about the order in which we did things?
  - What happened at the meeting?

3. Acceptability of content (15 min)

- What was it like to talk about cancer screening at the meeting?
  - How did you feel?
  - Can you remember what you thought of it at the time?
- How do you feel about talking about your faith and cancer screening?
  - What was it like?
  - What did you think of it at the time?
- Is there anything else you would have liked to talk about at the meeting?
  - Is there anything else you want to know about cancer screening?
  - Is there anything else you want to know about your faith and your health?
  - Is there anything else we should tell people about their faith and cancer screening?

4. Pre-workshop communications

- Before coming to the workshop, what did you think it was going to be like?
  - What did you think that the workshop was going to be about?
  - What did you hope to get from the workshop?
  - Did you feel that the workshop matched your expectations? How?
  - Did anything surprise you? explain.
- Before coming to the workshop, how do you feel about the information you were given?
  - Did you feel you had enough information? Why (not)?
  - Is there any other information that you would have liked? Explain.
  - What would you change about the information given?
- Can you tell me how hard or easy it was for you to come to the workshop? (burden)
  - Was it hard for you to travel to the venue [location]/join online?
  - Can you explain what made it hard/easy for you?
  - Is there anything that we could have done to make it better for you to join us?
- Was attending the workshop convenient to you? why (not)? (opportunity cost)
  - How did you feel about traveling to the venue?
  - Did you have to give up other commitments to attend the workshop? tell me how.
  - Can you tell me if attending the workshop was costly to you? can you tell me more.

5. Acceptability of delivery (20 min)

- In the first part of the workshop, you had a discussion with other Muslim women about cancer screening. How do you feel about talking with other women about cancer screening?
  - How did you feel during the meeting?
  - How did you feel before the meeting?
  - What did you think?
- How do you feel about the GP’s [insert name] talk about cancer screening??
  - What did you like about her talk?
  - Was there anything that didn’t go so well?
  - Is there anything else the GP should have explained or talked about?
  - How do you feel about how the GP presented the information about cancer screening? (self-efficacy)
- How do you feel about the Alimah’s talk about your faith and cancer screening?
  - What did you like about her talk?
  - Was there anything that didn’t go so well?
  - Is there anything else the Alimah should have explained or talked about?
  - Was there anything in the Alimah’s talk that was not clear? Explain (self-efficacy)
- How do you feel about the video of Fakhra’s screening experience we showed you at the start?
  - What did you like about the video?
  - Is there anything you didn’t like about the video?
  - Is there anything about the video that we should change?
- How do you feel about the video about cervical cancer screening ?
  - What did you like about the video?
  - Is there anything you didn’t like about the video?
  - Is there anything about the video that we should change?
- Is there anyone else you would have liked to be at the meeting?
  - Who would you like to talk to about your faith and cancer screening?
  - Who do you usually speak to for advice about your faith?
  - Who do you usually speak to for advice about cancer screening?
- Now we talked about the different parts of the workshop, can you tell me about what you think of the workshop as a whole? (coherence)
  - Do you understand how the workshop aims to help Muslim women to go to screening?
  - How do you feel about the order of the different parts?
  - Do you feel having a GP and an Alimah at the workshop was good? Why (not)?
  - Is there anything that we needed to change?
- Do you feel that the workshop was suitable for Muslim women? Please explain. (ethicality)
  - What made it (not) suitable?
  - As a Muslim woman, which part you liked the most?
  - Was there a part that was inappropriate? How?
  - What could we do to improve?
  - Was there anything that we could have done differently?
- How did you feel about the language of the workshop? (self efficacy)
  - How did you feel about how the translation was done?
  - What you would have liked to be different about the translation?
- How do you feel about the time/length of the workshop?
  - Do you feel that 2 hours were enough for the workshop?why?
  - What would you change about the length of the workshop?
  - Is there any part of the workshop that you felt needed more time? Explain.
- How do you feel about cancer screening after our meeting? (perceived effectiveness)
  - What did you learn at our meeting that you didn’t know before?
  - Have your feelings changed in any way after our meeting?
  - Do you think our meeting could change women’s minds about screening?
  - Do you think our meeting could change your mind about screening?
- Do you feel that our workshop will help you go to screening in the future? How? (perceived effectiveness)
  - Do you have any plans about going to screening after the workshop?
  - Do you feel that you can make a screening appointment?
  - Why do you feel that you can(not) go to screening?
  - What was most helpful?
- We gave you questionnaires before and after the workshop. What did you think of the questionnaires? How did you feel about filing these in? (self-efficacy)
  - How did you feel about how/the way these questionnaires were given? Online/paper?
  - Did you feel you had enough time to fill in the questionnaires?

6. Logistics:

- How did you feel about the venue [enter name] where we delivered the intervention?
  - What did you like (or didn’t like) about this venue?
  - What would you change about the venue?
  - Would you have preferred a different venue for the workshop? why?
- How did you feel about the break we had?
  - Did you feel we had enough time for the break? Why
  - How do you feel about the drinks/snacks on offer during the break?
  - What would you change about it?
- How do you feel about the lunch that was provided after the workshop?
  - What did you like (or didn’t like) about it?
  - What would you change?

7. Closing (10 min)

- Is there anything else you would like to add?
  - Anything else you would like to tell us about the meeting?
  - Is there anything we didn’t talk about that we should have asked about?
